# Paraspinal muscle quality in chronic low back pain: a systematic review and meta-analysis of muscle atrophy and fat infiltration

**DOI:** 10.1007/s00586-025-09454-z

**Published:** 2025-10-28

**Authors:** Ioannis Kalli, Marili Niglas, Maryam Kazemi Naeini, Maxim Freidin, Louise Thomas, Cristina Menni, Frances Williams

**Affiliations:** 1 King’s College London, London, UK; 2 University of Westminster, London, UK; 3 University of Milan, Milan, Italy

**Keywords:** Chronic low back pain, Paraspinal muscles, Cross-sectional area, Fat infiltration, Muscle atrophy, Lumbar spine

## Abstract

**Background:**

Chronic low back pain (cLBP) is the leading cause of disability worldwide, yet the specific musculoskeletal mechanisms underlying symptom persistence remain poorly understood. Structural changes in the spine-supporting paraspinal muscles, particularly the multifidus, erector spinae, and psoas major, have been implicated in its pathophysiology, but current evidence remains inconclusive.

**Purpose:**

To summarise and quantify differences in lumbar paraspinal muscle morphology and composition—specifically total cross-sectional area (tCSA), functional cross-sectional area (fCSA), and fat infiltration (FI)—between individuals with cLBP and asymptomatic controls, with stratification by vertebral level to assess segmental specificity.

**Study design/setting:**

Systematic review and meta-analysis.

**Methods:**

A comprehensive literature search of electronic databases was conducted. Twenty-one studies were included in the qualitative synthesis, and 10 met criteria for meta-analysis. Standardised mean differences (SMDs) were calculated using random-effects models, and subgroup analyses were performed by vertebral level. Sensitivity analyses assessed the impact of study quality and methodological factors.

**Outcome measures:**

Imaging-derived assessments of paraspinal muscle tCSA, fCSA, and FI.

**Results:**

Individuals with cLBP exhibited significantly lower multifidus tCSA and fCSA, alongside higher FI, particularly at mid-to-lower lumbar levels (L3/L4 to L5), indicating segment-specific muscle atrophy. These findings were generally robust across sensitivity analyses, though some were influenced by demographic and methodological variability. In contrast, differences in erector spinae and psoas major were less consistent, often confined to isolated lumbar levels or revealed only after sensitivity testing. High between-study heterogeneity was attributed to differences in imaging modality, muscle segmentation techniques, and participant characteristics.

**Conclusions:**

Multifidus demonstrates the most consistent structural alterations among paraspinal muscles in individuals with cLBP, reinforcing its potential relevance in diagnosis and the development of targeted rehabilitation strategies. Future research should prioritise standardised imaging protocols and reporting practices to reduce heterogeneity and improve clinical translation.

**Supplementary Information:**

The online version contains supplementary material available at 10.1007/s00586-025-09454-z.

## Introduction

Low back pain (LBP) is the leading cause of disability worldwide, responsible for more years lived with disability than any other condition across all age groups [1–3]. Clinically defined as pain or discomfort localised between the 12th rib and the inferior gluteal folds, with or without referred symptoms [4], LBP represents a complex, multifactorial condition shaped by the interplay of biomechanical, physiological, psychological, and social factors. Although its prevalence increases with age, LBP is not an inevitable consequence of ageing, with several modifiable risk factors including smoking, obesity, and physical inactivity, associated with its development and chronicity [5–8].

LBP is commonly classified based on its underlying aetiology as either specific or non-specific, with the latter accounting for approximately 90% of the cases, where no obvious anatomical source is identified [9]. Additionally, LBP is categorised according to symptom duration, with acute LBP lasting less than 6 weeks, subacute LBP persisting between 6 and 12 weeks, and chronic LBP extending beyond 3 months [10]. Notably, approximately 5–10% of individuals with LBP progress to chronic low back pain (cLBP), which imposes a substantial socioeconomic burden, including increased healthcare costs, prolonged work absenteeism, and a significant decline in health-related quality of life [11–13].

Growing research interest has focused on understanding the musculoskeletal factors contributing to cLBP, particularly the role of paraspinal muscles in the development and persistence of LBP. The multifidus, erector spinae, and psoas major muscles exhibit distinct anatomical and functional characteristics at different lumbar levels, with each contributing uniquely to spinal stability and intervertebral motion control [14, 15]. Structural changes within these muscles such as reduced cross-sectional area (CSA) and increased fat infiltration (FI), have been implicated in the pathophysiology of cLBP [14–18]. Muscle atrophy, characterised by a reduction in CSA, compromises spinal support, whereas FI, defined as the pathological replacement of contractile muscle with adipose tissue, has been suggested to impair muscle contractility, promote inflammation, and further destabilise the spine [19–22]. Such morphologic and compositional changes are commonly evaluated through non-invasive imaging modalities, including magnetic resonance imaging (MRI), computed tomography (CT), and ultrasound (US) [19–21, 23]. However, the clinical significance of these structural alterations remains poorly understood.

Several studies have investigated differences in paraspinal muscle morphology and composition between individuals with cLBP and asymptomatic controls, but their findings remain inconsistent. While some studies report significantly smaller CSA and increased FI in individuals with cLBP, others fail to identify substantial differences between groups [19–21, 23–26]. This variability in findings has led to the need for high-quality evidence synthesis to determine whether consistent morphological and compositional changes exist in individuals with cLBP.

Six systematic reviews have explored the relationship between paraspinal muscle characteristics and LBP [27–32]. However, two of these exclusively examined a specific spinal pathology, such as lumbar disc herniation and neuro-compressive disorders, limiting the applicability of their findings to the broader population with non-specific LBP, which constitutes the majority of the cases [28, 29]. The other four suggest a reduction in multifidus CSA among individuals with LBP, particularly at lower lumbar levels [27, 30–32]. However, these findings encompass heterogeneous populations spanning acute, subacute, and chronic pain phases without differentiation, and employ analytical approaches that average muscle data across vertebral levels. As acknowledged in the most recent systematic review [32], which used averaged values across all lumbar vertebral levels in its meta-analysis, this approach, while methodologically appropriate given data availability constraints, may obscure segment-specific adaptations that could provide critical insights into cLBP pathophysiology. In contrast, findings related to changes in the erector spinae and psoas major muscles remain conflicting, with some reviews reporting no significant difference between individuals with LBP and controls [30–32]. Similarly, the relationship between paraspinal muscle FI and LBP remains unclear, as some studies attribute increased fat content to aging and disuse rather than LBP itself [30–32].

To date, no systematic review has exclusively examined paraspinal muscle morphology and composition in cLBP populations using segment-specific analytical approaches. This represents a significant gap in the literature, given the distinct anatomical, biomechanical, and functional characteristics of paraspinal muscles across lumbar levels, and the potential for cLBP to involve regionally-specific rather than uniform muscle adaptations. Therefore, this systematic review aims to synthesise and quantify differences in lumbar paraspinal muscle morphology and composition, specifically tCSA, fCSA, and FI, between individuals with cLBP and asymptomatic controls, with stratification by vertebral level to identify segment-specific patterns that may inform targeted diagnostic and therapeutic strategies.

## Methods

### Protocol and registration

This systematic review was conducted in accordance with the Preferred Reporting Items for Systematic Reviewers and Meta-analysis (PRISMA) 2020 guidelines [33]. The protocol was registered with the International Prospective Register of Systematic Reviews (PROSPERO; CRD420250650415) on February 27, 2025, and is accessible at: https://www.crd.york.ac.uk/PROSPERO/view/CRD420250650415.

### Search strategy and study selection

A comprehensive literature search was performed across four electronic databases: MEDLINE, EMBASE, PubMed, and Scopus, covering publications up to January 31, 2025. Search strategies were tailored to each database and are presented in Supplementary Fig. 1. All retrieved citations were imported into EndNote 21 for reference management, and duplicates were removed.

Title and abstract screenings were conducted independently by one reviewer (IK), with full-text articles subsequently assessed by two reviewers (IK and FW) to determine final eligibility. Discrepancies during full-text screening were resolved through consensus.

### Study selection criteria

Studies included in this systematic review were selected based on predefined inclusion and exclusion criteria.

The inclusion criteria were as follows: cohort, case-control, or cross-sectional studies published in English; studies involving adults (≥ 18 years old) diagnosed with chronic low back pain (cLBP), defined as pain persisting for ≥ 3 months; studies including asymptomatic control participants with no history of low back pain (LBP) or spinal disorders; studies assessing at least one muscle morphology feature, such as muscle cross-sectional area or fat infiltration; studies assessing at least one of the following paraspinal muscles: multifidus, erector spinae, or psoas major; and studies utilising at least one of the following imaging techniques: magnetic resonance imaging (MRI), computed tomography (CT), or diagnostic ultrasound (DUS).

Studies were excluded if they involved individuals with prior lumbar spine surgery; focused on low back pain secondary to fracture, malignancy, infection, or autoimmune disease; included individuals with structural spinal abnormalities (e.g., scoliosis, lumbar degenerative kyphosis); specifically recruited athletes or individuals with occupational exposures; or were animal studies.

**Fig. 1:**
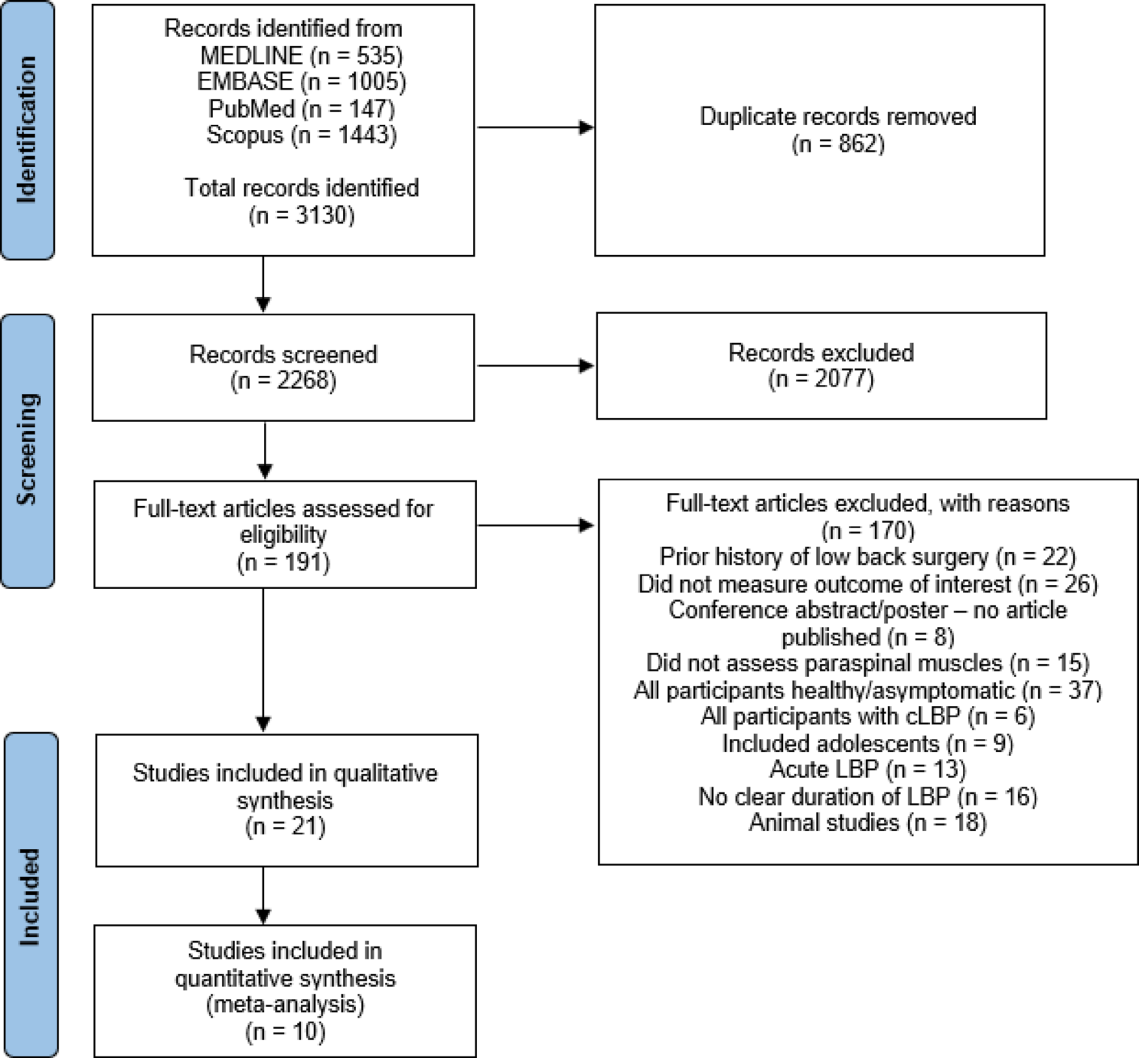
Flow diagram for the study selection process

No minimum sample size threshold was applied as an exclusion criterion. This decision was made because of the relatively limited number of eligible studies in this field, and applying a sample size cut-off would have risked omitting potentially informative evidence. Detailed reasons for exclusion were documented using a standardised template to ensure transparency and consistency in the review process (Fig. 1).

### Quality assessment

The methodological quality of the included case-control studies was independently assessed by two reviewers (IK and MN) using the Newcastle–Ottawa Scale (NOS), a validated tool designed to evaluate the quality of nonrandomised studies [34]. For cross-sectional studies, an adapted version of the NOS was applied, consistent with approaches used in previous systematic reviews and as detailed in the Supplementary Material. The NOS framework evaluates three domains—selection, comparability, and outcome/exposure—with a maximum possible score of 9 points (up to 4 for selection, 2 for comparability, and 3 for outcome/exposure).

Studies were classified as:

- Good quality: 3 or 4 stars in selection domain and 1 or 2 stars in comparability domain and 2 or 3 stars in outcome/exposure domain.
- Fair quality: 2 stars in selection domain and 1 or 2 stars in comparability domain and 2 or 3 stars in outcome/exposure domain.
- Poor quality: 0 or 1 star in selection domain or 0 stars in comparability domain or 0 or 1 stars in outcome/exposure domain.

Inter-rater reliability was assessed using Cohen’s kappa coefficient (κ) [35].

Any discrepancies in the selection process between the two reviewers were resolved through open discussion; if consensus was not reached, a third reviewer (FW) was consulted.

### Data extraction

A single reviewer (IK) extracted data using a standardised Microsoft Excel form developed specifically for this review (Table 1). The data extraction included study characteristics, participant demographics, imaging modality, target muscle(s), disc level(s), and outcome measures (means, standard deviations, and sample sizes). Extracted data were cross verified by a second reviewer (FW). Any inconsistencies were resolved through discussion.

**Table 1:** Characteristics of studies examining the relationship between paraspinal muscle morphology and cLBP

Study	Design	Participant characteristics	Imaging tool	Disc level	Muscle	Outcome measure(s)	Findings
Mengiardi et al. [40]	Case-control	*N*= 25 cLBP (40.9 yrs, BMI 23.6 kg/m²) *N* = 25 controls (39.8 yrs, BMI: 23.7 kg/m²)	MRI, T2	L4/L5	MF	FC	↑ MF FI in cLBP
Hides et al. [23]	Case-control	*N* = 42 cLBP (46.8 ± 13.2 yrs) *N* = 40 controls (28.4 ± 5.7 yrs)	US	L2-L5	MF	tCSA	L4 & L5: ↓ MF tCSA in cLBP
Wallwork et al. [41]	Case-control	*N* = 11 cLBP (33.9 yrs) *N* = 11 controls (33.4 yrs)	US	L2-L5	MF	tCSA	L2: ↑ MF tCSA in cLBP L5: ↓ MF tCSA in cLBP
Arbanas et al. [42]	Case-control	*N* = 42 cLBP (Mean age: 51.1 ± 14.5 yrs, Mean BMI: 26.6 ± 4.4 kg/m²) *N* = 49 controls (Mean age: 55.1 ± 17.1 yrs, Mean BMI: 26.6 ± 5.2 kg/m²)	MRI, T2	L3/L4 L4/L5 L5/S1	PM	tCSA FI	L3/L4 & L4/L5: ↑ PM tCSA in cLBP
Singh et al. [43]	Case-control	*N* = 50 cLBP (Mean age: 33.54 ± 8.33 yrs, Mean BMI: 25.12 ± 2.67 kg/m²) *N* = 15 controls (Mean age: 28.93 ± 7.24 yrs, Mean BMI: 24.69 ± 2.67 kg/m²)	MRI, T2	L3/L4 L4/L5 L5/S1	MF ES PM	tCSA	Trend toward ↓ tCSA in cLBP across all muscles
Sions et al. (a) [44]	Case-control	*N* = 57 cLBP (Mean age: 70.5 ± 6.8 yrs, Mean BMI: 29.6 ± 5.8 kg/m²) *N* = 49 controls (Mean age: 72.2 ± 6.6 yrs, Mean BMI: 27.3 ± 4.59 kg/m²)	MRI, T1	L5	MF	FI tCSA rmCSA	↑ MF FI in cLBP
Sions et al. (b) [45]	Case-control	*N* = 53 cLBP (Mean age: 69.9 yrs, Mean BMI: 28.8 kg/m²) *N* = 43 controls (Mean age: 72.2 yrs, Mean BMI: 27.3 kg/m²)	MRI, T1	L2-L5	MF ES PM	From all levels: Mean FI Mean rmCSA	↑ Mean MF FI in cLBP ↓ Mean ES rmCSA in cLBP
Prasetyo et al. [56]	Cross-sectional	*N* = 24 cLBP (Median age: 35.5 yrs, Median BMI: 20.6 kg/m²) *N* = 20 controls (Median age: 28 yrs, Median BMI: 22.9 kg/m²)	CT scan	L4 L5	MF	Median FI ratio	L4: ↑ MF FI in cLBP L5: ↑ MF FI in cLBP
Ballatori et al. [46]	Case-control	*N* = 39 cLBP (Mean age: 47.43 ± 12.31 yrs, Mean BMI: 25.70 ± 5.08 kg/m²) *N* = 18 controls (Mean age: 42.74 ± 12.81 yrs, Mean BMI: 24.05 ± 4.34 kg/m²)	MRI, T1, T2	L1/L2 - L5/S1	MF	FF	L3/L4: ↑ MF FF in cLBP
Huang et al. [25]	Case-control	*N* = 60 cLBP (Mean age: 46.32 ± 16.99 yrs, Mean BMI: 21.71 ± 1.16 kg/m²) *N* = 32 controls (Mean age: 44.87 ± 17.59 yrs, Mean BMI: 21.75 ± 1.23 kg/m²)	MRI, T1, T2	L4/L5 L5/S1	MF ES	tCSA PDFF	L4/L5: ↓MF tCSA in cLBP L4/L5 & L5/S1: ↑ ES and MF PDFF in cLBP
Sakai et al. [47]	Case-control	*N* = 203 cLBP (Mean age: 79.00 ± 6.04 yrs, Mean BMI: 23.92 ± 3.77 kg/m²) *N* = 203 controls (Mean age: 78.96 ± 5.98 yrs, Mean BMI: 24.07 ± 3.63 kg/m²)	MRI, T2	L1/L2 L4/L5	MF ES	tCSA	L1/L2 & L4/L5: ↓ trunk muscle average tCSA in cLBP
Eksi et al. [26]	Cross-sectional	*N* = 66 cLBP *N* = 66 controls Mean age for all: 37.57 ± 8.01 yrs	MRI, T1, T2	L1/L2 - L5/S1	MF ES PM	FI	L1/L2 - L5-S1: ↑ MF and ES FI in cLBP L4-L5 & L5/S1: ↑ PM FI in cLBP
Giordan et al. [14]	Case-control	*N* = 153 cLBP (Median age: 56 years, Median BMI: 33.6 kg/m²) *N* = 52 controls (Median age: 50 years, Median BMI: 25 kg/m²)	MRI, T1, T2	L1/L2 - L5/S1	MF ES PM	From all levels: Mean tCSA Mean FI	↓ Mean MF, ES and PM in cLBP
Yazici et al. [48]	Cross-sectional	*N* = 35 CNLBP (Mean age: 36.97 ± 13.6 yrs, Mean BMI: 25.84 ± 5.61 kg/m²) *N* = 38 LDH (Mean age: 37.92 ± 10.57 yrs, Mean BMI: 26.49 ± 5.14 kg/m²) *N* = 36 controls (Mean age: 36.36 ± 12.12 yrs, Mean BMI: 25.71 ± 3.63 kg/m²)	MRI, T1, T2	L3/L4 - L5/S1	MF ES	From all levels: Mean tCSA Mean TCSA Mean FI	↑ Mean right & left MF & ES tCSA in cLBP ↓ Mean left ES tCSA in cLBP
Chen et al. [50]	Cohort	*N* = 100 cLBP with LDH (Mean age: 52.10 ± 8.82 yrs, Mean BMI: 27.66 ± 5.65 kg/m²) *N* = 120 acute LBP with LDH, Mean age: 51.25 ± 9.72 yrs, Mean BMI: 28.21 ± 5.45 kg/m²) *N* = 80 controls (Mean age: 53.21 ± 10.36 yrs, Mean BMI: 27.92 ± 4.32 kg/m²)	MRI, T1, T2	L4/L5	MF	FI tCSA fCSA	↓ MF tCSA in cLBP
Liu et al. [50]	Case-control	*N* = 136 cLBP (Mean age: 41.43 ± 10.54 yrs, Mean BMI: 23.70 ± 3.08 kg/m²) *N* = 119 controls (Mean age: 40.82 ± 12.70 yrs, Mean BMI: 23.07 ± 2.31 kg/m²)	MRI, T2	L1/L2 - L5/S1	MF ES	tCSA fCSA FI	L5/S1: ↑ ES tCSA in cLBP L4/L5: ↓ ES fCSA in cLBP L4/L5 & L5/S1: ↑ MF and ES FI in cLBP
Luo et al. [51]	Case-control	*N* = 56 Unilateral NCLBP (Mean age: 25.70 ± 1.96 yrs, Mean BMI: 21.98 ± 3.77 kg/m²) *N* = 51 controls (Mean age: 25.33 ± 2.02 yrs, Mean BMI: 21.02 ± 3.00 kg/m²)	MRI, T1, T2	L1 - L5	MF ES	FF	L2 – L5: ↑ MF FF in cLBP
Mahmoudi et al. [52]	Case-control	*N = 15 CNLBP with clinical LSI (Mean age: 34.4 ± 7.33 yrs, Mean BMI: 26.99 ± 1.3 kg/m²) *N* = 15 CNLBP without clinical LSI (Mean age: 38.4 ± 6.16 yrs, Mean BMI: 27.01 ± 1.25 kg/m² *N* = 15 controls (Mean age: 34.13 ± 4.92 years, Mean BMI: 26.06 ± 1.93 kg/m²)	MRI, T2	L2/L3 - L5/S1	MF ES PM	rCSA rmCSA FI	L3/L4: ↓ MF rmCSA & MF FI in cLBP L4/L5 & L5/S1 ↓ MF rmCSA in cLBP L4/L5 & L5-S1: ↑ MF FI in cLBP L4/L5*: ↑ ES rmCSA in cLBP
Mamatha et al. [53]	Cohort	*N* = 50 cLBP patients *N* = 50 controls	MRI	L4	MF ES PM	tCSA	↓ MF tCSA in cLBP ↑ ES tCSA in cLBP
Newell et al. [54]	Case-control	Female cLBP: *N* = 20 (Mean age: 45.50 ± 10.07 yrs, Mean BMI: 26.39 ± 2.84 kg/m²) Female controls: *N* = 16 (Mean age: 63.31 ± 13.70 yrs, Mean BMI: 22.77 ± 7.00 kg/m²) Male cLBP: *N* = 16 (Mean age: 50.56 ± 18.41 yrs, Mean BMI: 27.71 ± 5.63 kg/m²) Male controls: *N* = 17 (Mean age: 55.82 ± 15.11 yrs, Mean BMI: 21.67 ± 2.25 kg/m²)	MRI, T2	L1/L2 - L5/S1	MF ES PM	tCSA fCSA FI	L4/L5: ↑ ES tCSA in cLBP
Yang et al. [55]	Case-control	*N* = 23 cLBP patients (Median age: 31.0 yrs, Median BMI: 22.72 kg/m²) *N* = 20 controls (Median age: 30.75 years, Median BMI: 22.48 kg/m²)	MRI, T1, T2	L1/L2 - L5/S1	MF ES PM	From all levels: Mean FC	↑ right MF FC & ES in cLBP

cLBP, chronic low back pain; CNLBP, chronic non-specific low back pain; LDH, lumbar disc herniation; LSI, lumbar segmental instability; US, ultrasound; CT, computed tomography; MRI, magnetic resonance imaging MF, multifidus; ES, erector spinae; PM, Psoas major; FC, fat content; tCSA, total cross-sectional area; FI, fat infiltration; rmCSA, relative muscular cross-sectional area; FF, fat fraction; PDFF, proton density fat fraction; fCSA, functional cross-sectional area; rCSA, relative cross-sectional area

### Data synthesis and analysis

Quantitative data extracted from studies examining cLBP were systematically categorised according to outcomes for three key paraspinal muscles—multifidus, erector spinae, and psoas major—across three anatomical and compositional parameters: tCSA, fCSA, and FI. For each study, the standardised mean difference (SMD) and corresponding 95% confidence intervals (CIs) were calculated from the reported sample size, mean, and standard deviation, thereby enabling direct comparisons between continuous muscle measurements despite heterogeneity in both study design and measurement techniques [36]. Cohen’s conventional thresholds were applied as ranges: SMD values of 0.2 to < 0.5 were considered small effects, 0.5 to < 0.8 medium effects, and ≥ 0.8 large effects [36]. Effect sizes were considered statistically significant when 95% CIs did not include zero, with negative SMDs indicating lower mean values in the cLBP group compared to controls.

Separate random-effects meta-analyses were conducted for each muscle and outcome parameter using the restricted maximum likelihood (REML) method [37], which accounts for between-study variability attributable to differences in imaging modalities, segmentation approach, and sample characteristics. To explore level-specific differences in muscle morphology and composition between individuals with cLBP and asymptomatic controls, analyses were further stratified by vertebral level. Subgroup meta-analyses were only conducted when two or more independent studies contributed data at a given level. Studies that lacked essential quantitative data (i.e., sample size, mean, or standard deviation) were excluded from the meta-analysis.

Heterogeneity across studies was assessed using Cochrane’s Q test and the I² statistic, with thresholds for interpretation as follows: 0%–24% = low, 25%–49% = moderate, 50%–74% = substantial, and 75%–100% = considerable heterogeneity [38]. Sensitivity analyses were performed using the jackknife (leave-one-out) method for each muscle and vertebral level where at least three studies were available, in order to examine the robustness of pooled estimates and identify potential outliers or influential studies [39].

## Results

### Study selection

The systematic search yielded a total of 3,130 records. After removing duplicates, 2,268 unique articles remained for title and abstract screening. Of these, 191 full-text articles were retrieved for detailed evaluation. Following the application of predefined eligibility criteria, 170 studies were excluded with reasons. Ultimately, 21 studies met the inclusion criteria for the qualitative synthesis, of which 10 studies provided sufficient quantitative data for inclusion in the meta-analysis (Fig. 1).

### Methodological assessment

The quality of the 21 studies included in qualitative synthesis was evaluated using the NOS scale (Table 2). Based on NOS scoring criteria, 20 studies were rated as ‘good’ quality [14, 23, 25, 26, 40–55], while one study was classified as ‘poor’ [56]. The overall inter-rater agreement for quality assessment was high, as reflected by Cohen’s kappa coefficient, κ = 0.85, indicating substantial agreement between reviewers.

**Table 2:** The Newcastle-Ottawa scale (NOS) for study quality assessment

Study	Selection	Comparability	Exposure	Quality
Mengiardi et al. 2006	***	*	***	Good
Hides et al. 2008	***	*	**	Good
Wallwork et al. 2009	****	**	**	Good
Arbanas et al. 2013	***	*	***	Good
Singh et al. 2016	****	*	***	Good
Sions et al. 2017 (a)	****	**	**	Good
Sions et al. 2017 (b)	****	*	***	Good
Prasetyo et al. 2020	****	-	***	Poor
Ballatori et al. 2022	****	*	***	Good
Huang et al. 2022	****	*	***	Good
Sakai et al. 2022	***	*	**	Good
Eksi et al. 2023	***	*	***	Good
Giordan et al. 2023	***	*	**	Good
Yazici et al. 2023	***	*	***	Good
Chen et al. 2024	***	*	***	Good
Liu et al. 2024	****	*	**	Good
Luo et al. 2024	****	*	***	Good
Mahmoudi et al. 2024	****	*	***	Good
Mamatha et al. 2024	****	*	***	Good
Newell et al. 2024	****	*	**	Good
Yang et al. 2024	****	*	***	Good

### Subgroup meta-analysis

#### Multifidus

##### Total cross-sectional area (tCSA)

**Fig. 2:**
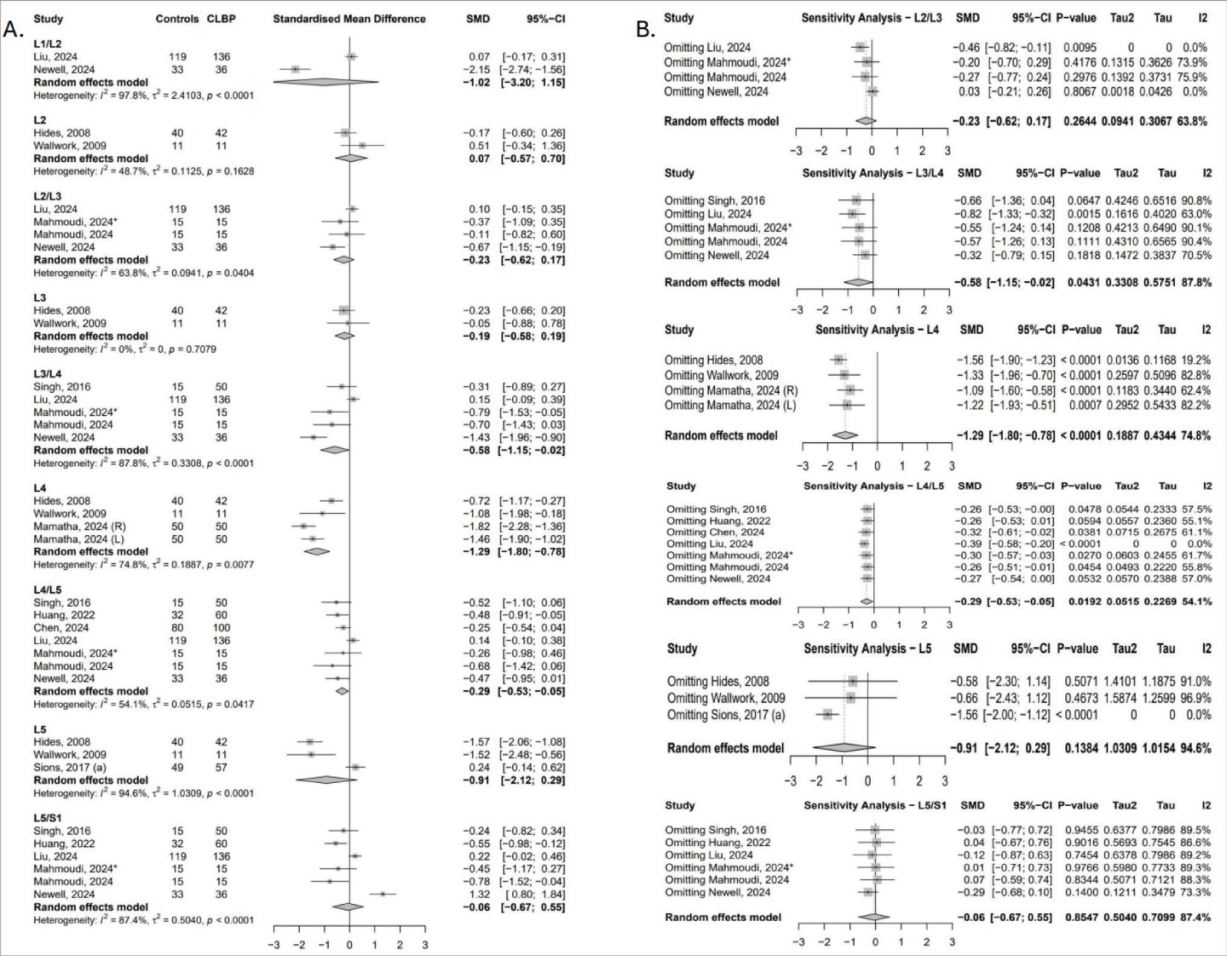
(**A**) Forest plot showing standardised mean difference in multifidus tCSA between individuals with cLBP and asymptomatic controls. (**B**) Leave-one-out sensitivity analysis assessing the influence of individual studies on the pooled estimates of multifidus tCSA across lumbar levels

Ten studies contributed data on multifidus tCSA across nine lumbar levels [23, 25, 41, 43, 44, 49, 50, 52–54]. Compared to asymptomatic controls, individuals with cLBP exhibited significantly lower tCSA at L3/L4 (SMD = − 0.58; 95% CI: − 1.15 to − 0.02; I² = 63.8%), L4 (SMD = − 1.29; 95% CI: − 1.80 to − 0.78; I² = 74.8%), and L4/L5 (SMD = − 0.29; 95% CI: − 0.53 to − 0.05; I² = 54.1%), reflecting greater muscle atrophy at the mid-to-lower lumbar segments (Fig. 2A). Sensitivity analyses confirmed the robustness of the L4 finding, while the effects at L3/L4 and L4/L5 were influenced by study-level variability, particularly the inclusion of data from Liu [50], Newell [54], and Huang [25]. At L2/L3 and L5, pooled effects were not statistically significant in the primary analysis but reached significance upon exclusion of Liu [54] and Sions [44], respectively (Fig. 2B).

##### Functional cross-sectional area (fCSA)

**Fig. 3:**
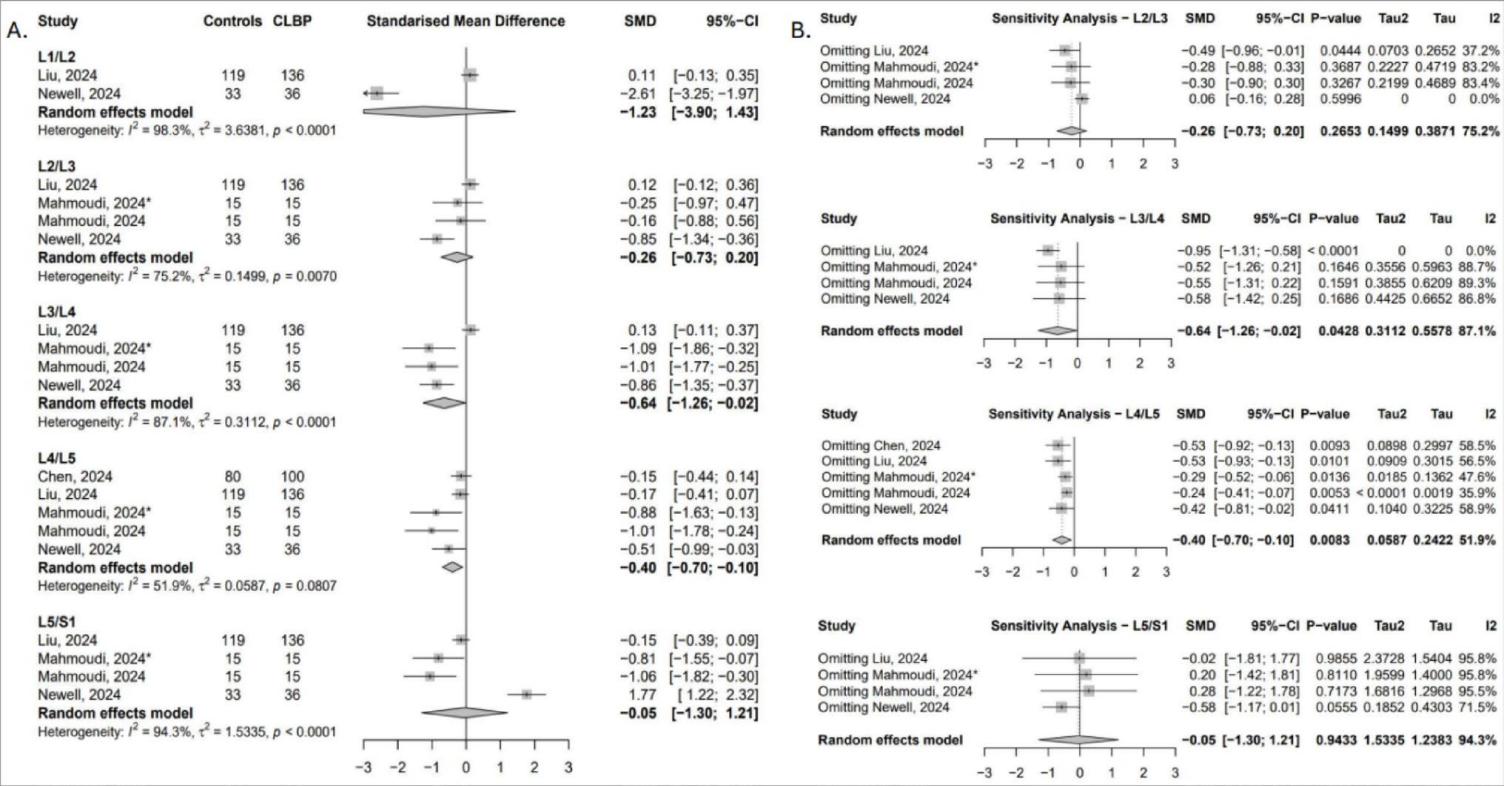
(**A**) Forest plot showing standardised mean difference in multifidus fCSA between individuals with cLBP and asymptomatic controls. (**B**) Leave-one-out sensitivity analysis assessing the influence of individual studies on the pooled estimates of multifidus fCSA across lumbar levels

Four studies reported multifidus fCSA across four lumbar levels [49, 50, 52, 54]. Compared to controls, individuals with cLBP showed significantly lower fCSA at L3/L4 (SMD = − 0.64; 95% CI: − 1.26 to − 0.02; I² = 87.1%) and L4/L5 (SMD = − 0.40; 95% CI: − 0.70 to − 0.10; I² = 51.9%), reflecting greater functional atrophy at these segments (Fig. 3A). Sensitivity analyses confirmed the robustness of the L4/L5 finding. At L3/L4, the pooled effect was highly sensitive to study inclusion; exclusion of Liu [50] strengthened the effect and eliminated heterogeneity, while removal of other studies rendered the result non-significant. At L2/L3, significance emerged only after omitting the study by Liu [50], whereas removal of Newell [54] reversed the direction of effect without reaching statistical significance (Fig. 3B).

##### Fat infiltration (FI)

**Fig. 4:**
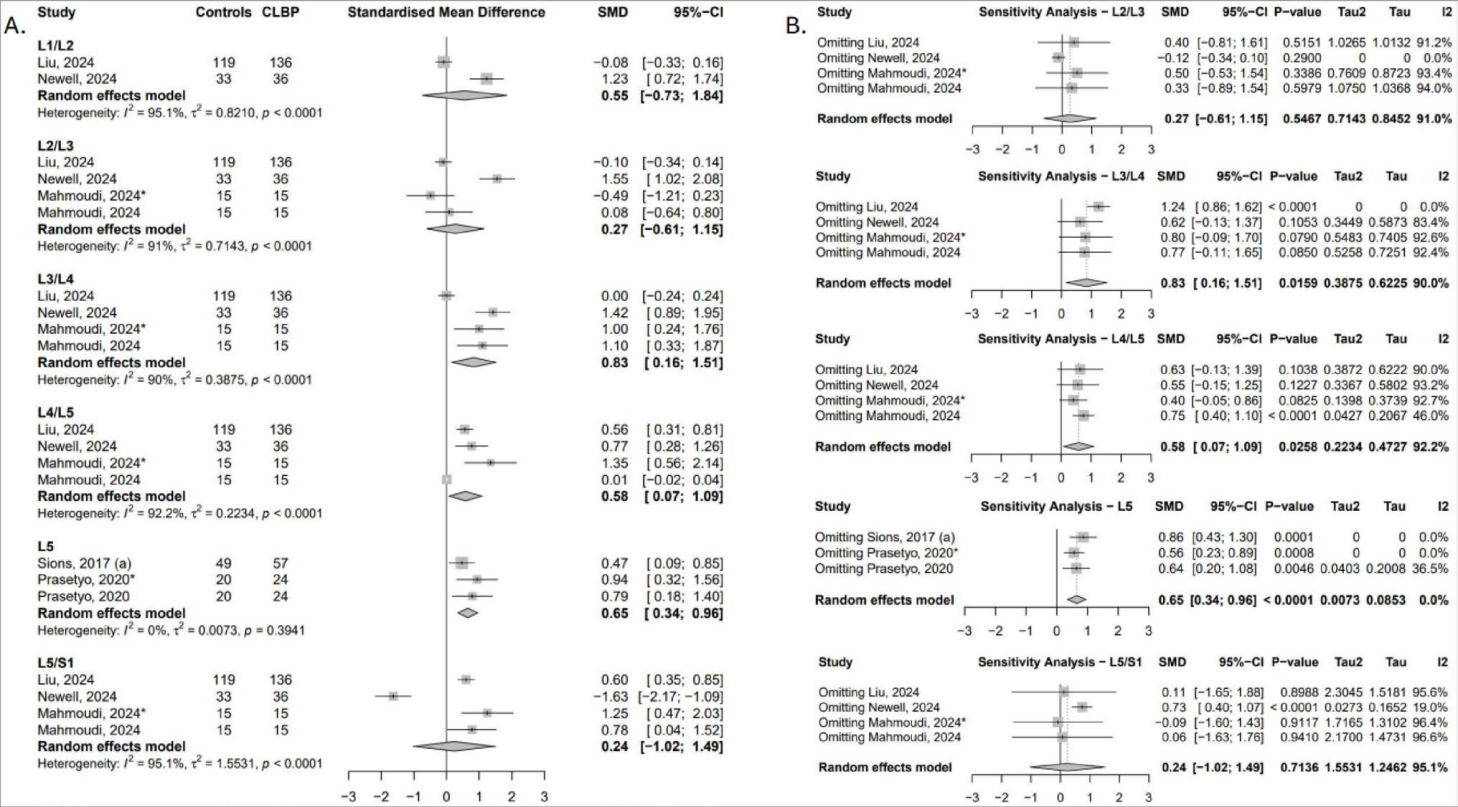
(**A**) Forest plot showing standardised mean difference in multifidus FI between individuals with cLBP and asymptomatic controls. (**B**) Leave-one-out sensitivity analysis assessing the influence of individual studies on the pooled estimates of multifidus FI across lumbar levels

Five studies assessed multifidus FI across six lumbar levels [44, 50, 52, 54, 56]. Significantly greater FI in cLBP patients was observed at L3/L4 (SMD = 0.83; 95% CI: 0.16 to 1.51), L4/L5 (SMD = 0.58; 95% CI: 0.07 to 1.09), and L5 (SMD = 0.65; 95% CI: 0.34 to 0.96), indicating increased fatty degeneration in mid-to-lower lumbar segments (Fig. 4A). The robustness of the findings at L5 were confirmed following the sensitivity analysis, however, findings at L3/L4 were highly sensitive to the inclusion of either the Newell [54] or Mahmoudi study [52]. The L4/L5 result was less stable, becoming non-significant in most iterations. At L5/S1, exclusion of the Newell study [54], revealed a significant effect, while exclusion of the lumbar instability subgroup from Mahmoudi et al. [52]., reversed the direction of association (Fig. 4B).

#### Erector spinae

##### Total Cross-Sectional area (tCSA)

**Fig. 5:**
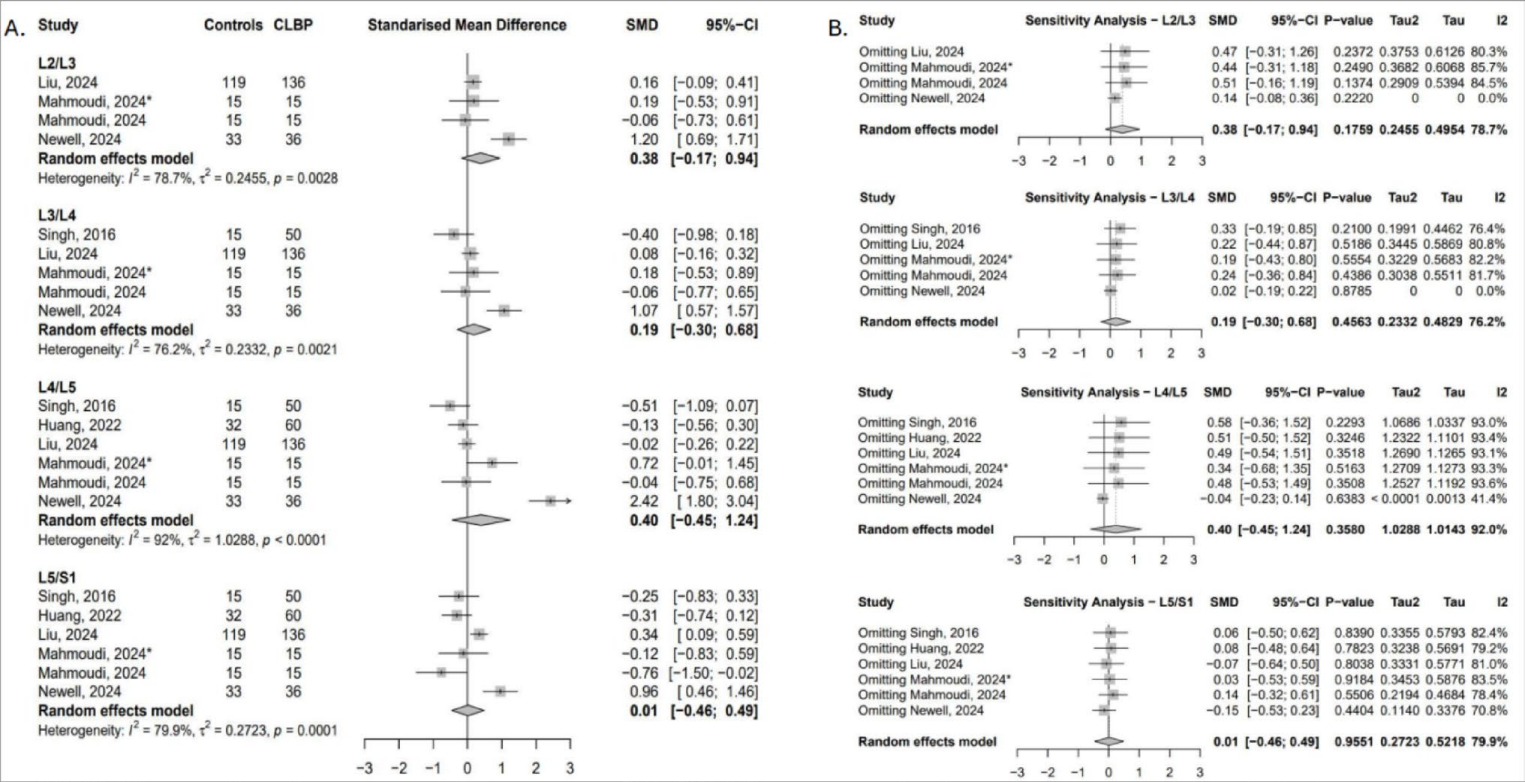
(**A**) Forest plot showing standardised mean difference in erector spinae tCSA between individuals with cLBP and asymptomatic controls. (**B**) Leave-one-out sensitivity analysis assessing the influence of individual studies on the pooled estimates of erector spinae tCSA across lumbar levels

Five studies provided data on erector spinae tCSA across four lumbar levels [25, 43, 50, 52, 54]. The meta-analysis revealed no statistically significant differences, although pooled effects suggested a non-significant trend toward greater erector spinae tCSA in cLBP patients (Fig. 5A). Sensitivity analyses revealed that exclusion of the Newell study [54] at L4/L5 and L5/S1 reversed the effect direction, a pattern also observed at L5/S1 with the exclusion of the Liu study [50] (Fig. 5B).

##### Functional cross-sectional area (fCSA)

**Fig. 6:**
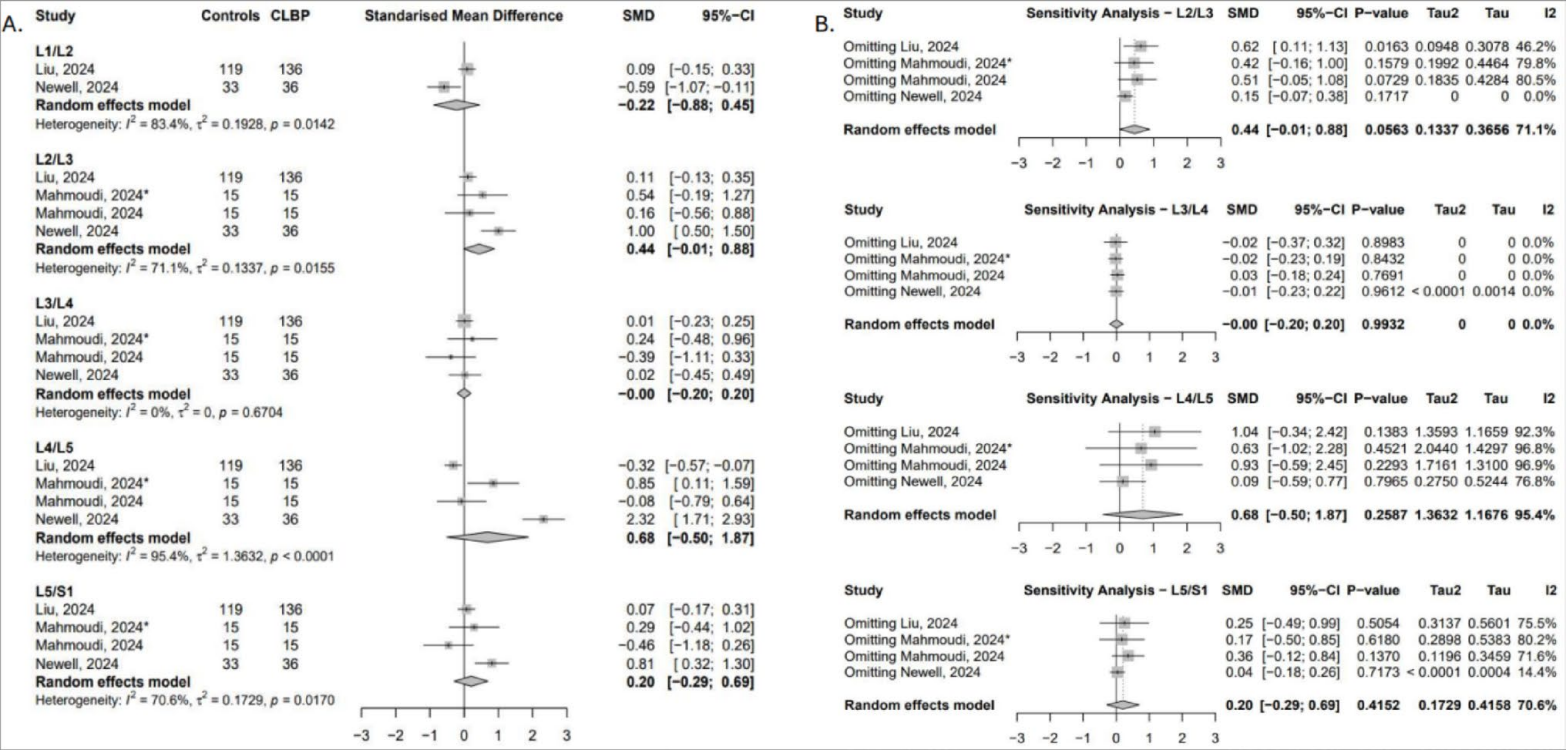
(**A**) Forest plot showing standardised mean difference in erector spinae fCSA between individuals with cLBP and asymptomatic controls. (**B**) Leave-one-out sensitivity analysis assessing the influence of individual studies on the pooled estimates of erector spinae fCSA across lumbar levels

Three studies provided data across five lumbar levels [50, 52, 54], but no statistically significant differences were observed at any level. A non-significant trend towards greater erector spinae fCSA in asymptomatic controls was observed at the lower lumbar levels (Fig. 6A). However, at L2/L3, exclusion of the Liu study [50] resulted in a statistically significant positive effect (SMD = 0.62; 95% CI: 0.11 to 1.13), indicating significantly greater erector spinae fCSA in controls at this level (Fig. 6B).

##### Fat infiltration (FI)

**Fig. 7:**
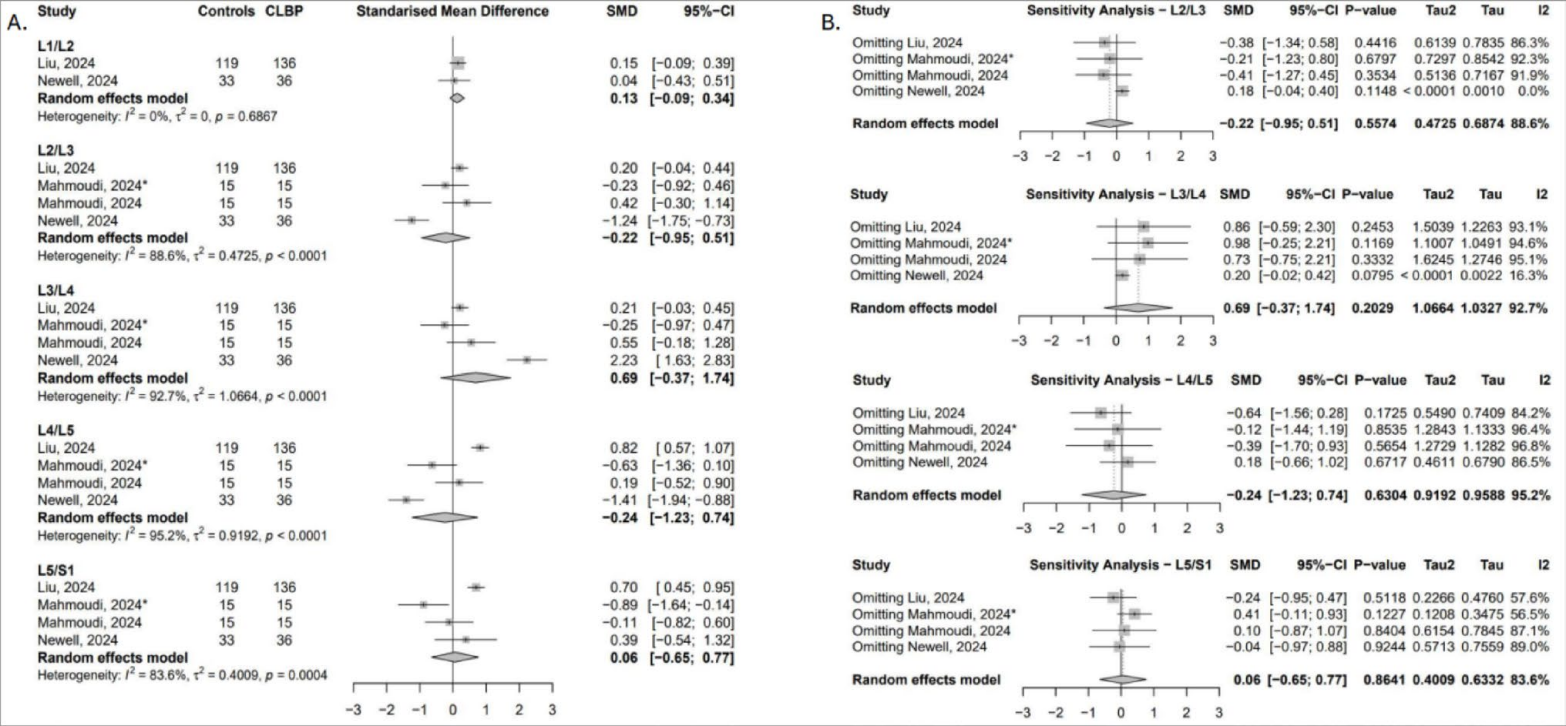
(**A**) Forest plot showing standardised mean difference in erector spinae FI between individuals with cLBP and asymptomatic controls. (**B**) Leave-one-out sensitivity analysis assessing the influence of individual studies on the pooled estimates of erector spinae FI across lumbar levels

Four studies contributed data across five lumbar levels [26, 50, 52, 54], but no statistically significant differences were identified, with the direction of effect estimates varying across levels (Fig. 7A). Sensitivity analyses revealed that exclusion of the Newell study [54] shifted the SMD at L2/L3 to a positive value, favouring higher FI in cLBP patients, although the result remained non-significant. In contrast, removal of either the Newell [54] or Liu study [50] at L5/S1 reversed the effect direction in favour of higher FI in controls, though these results also remained non-significant (Fig. 7B).

#### Psoas major

##### Total cross-sectional area (tCSA)

**Fig. 8:**
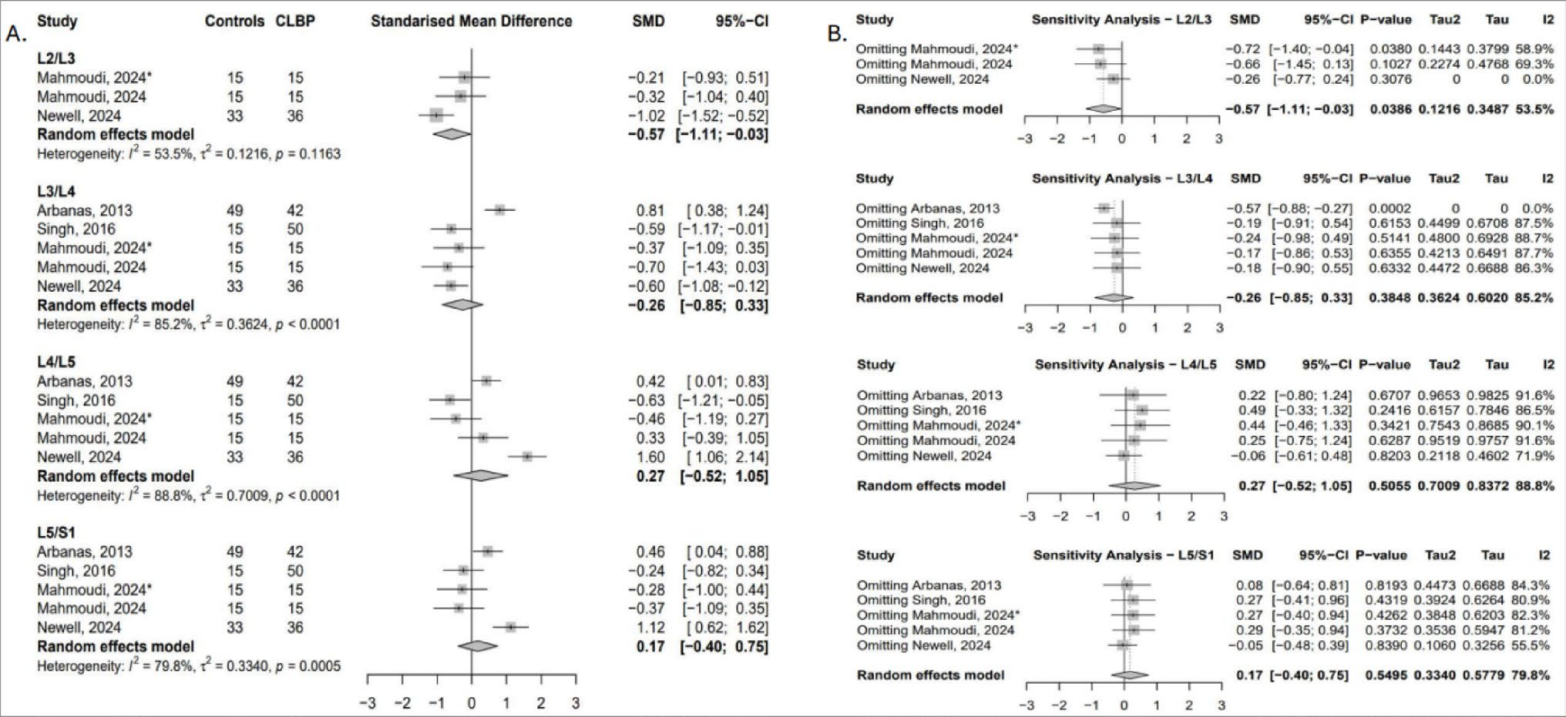
(**A**) Forest plot showing standardised mean difference in psoas major tCSA between individuals with cLBP and asymptomatic controls. (**B**) Leave-one-out sensitivity analysis assessing the influence of individual studies on the pooled estimates of psoas major tCSA across lumbar levels

Four studies provided data across four lumbar levels [42, 43, 52, 54], showing a significant reduction in psoas major tCSA at L2/L3 in cLBP patients (SMD = − 0.57; 95% CI: − 1.11 to − 0.03; I² = 53.5%) (Fig. 8A). This finding was sensitive to the exclusion of the Newell study [54] and Mahmoudi’s non-segmental instability subgroup [52]. At L3/L4, exclusion of Arbanas [42] resulted in a significantly negative pooled effect, suggesting higher tCSA in controls. At L4/L5 and L5/S1, excluding Newell’s study [54] shifted the effect toward smaller psoas major tCSA in cLBP patients, though these results remained non-significant (Fig. 8B).

##### Functional cross-sectional area (fCSA)

**Fig. 9:**
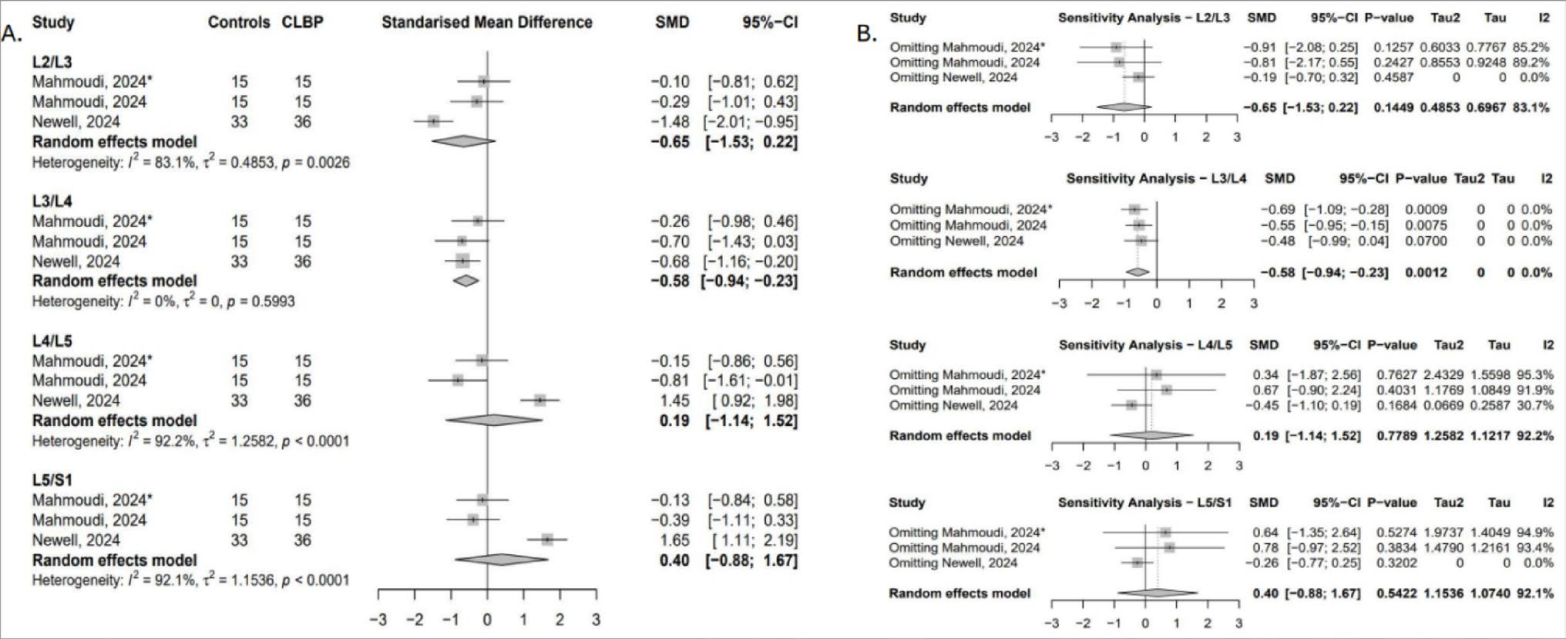
(**A**) Forest plot showing standardised mean difference in psoas major fCSA between individuals with cLBP and asymptomatic controls. (**B**) Leave-one-out sensitivity analysis assessing the influence of individual studies on the pooled estimates of psoas major fCSA across lumbar levels

Two studies reported data across four lumbar levels [52, 54]. A significant reduction in psoas major fCSA was found at L3/L4 in cLBP patients (SMD = − 0.58; 95% CI: − 0.94 to − 0.23; I² = 0%), indicating greater muscle atrophy at this level (Fig. 9A). This result was sensitive to exclusion of the Newell study [54]. At L4/L5 and L5/S1, removing Newell’s data [54] reversed the effect direction in favour of greater fCSA in controls, though results remained non-significant, with heterogeneity reduced to 30.2% and 0%, respectively (Fig. 9B).

##### Fat infiltration (FI)

**Fig. 10:**
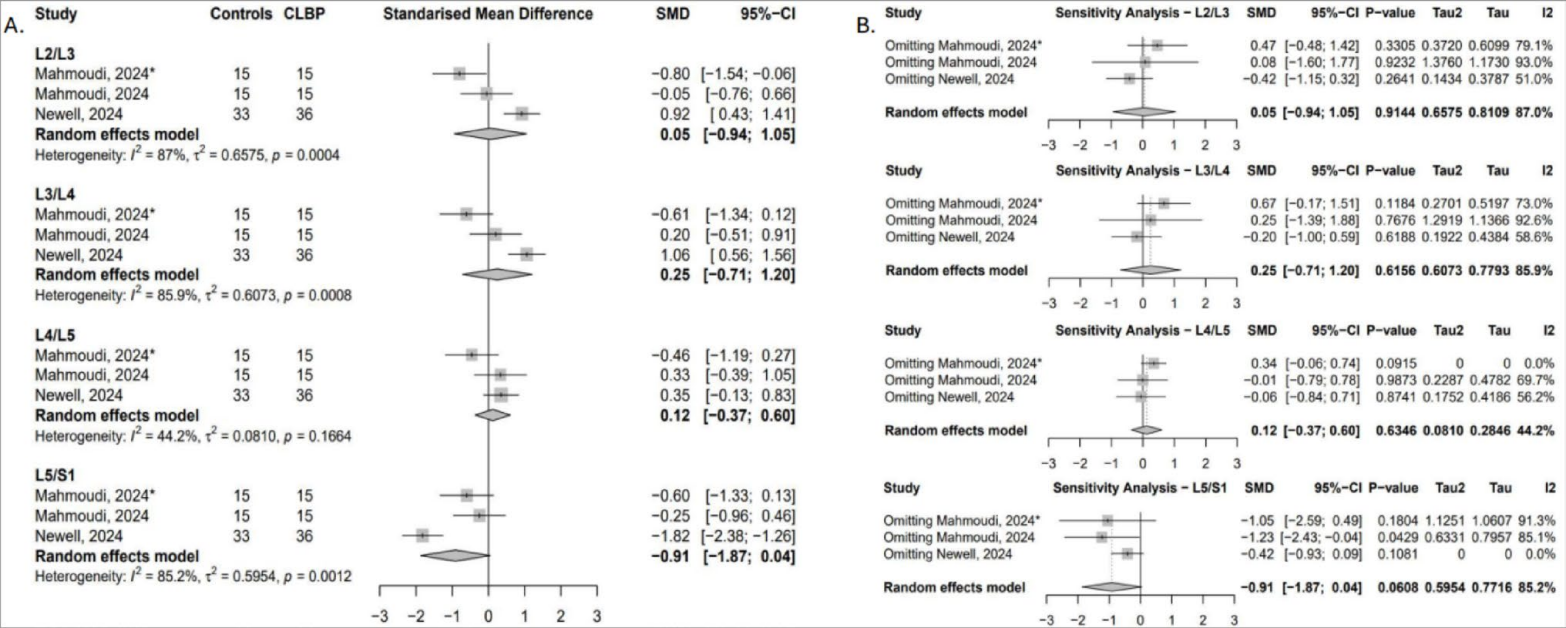
(**A**) Forest plot showing standardised mean differences in psoas major FI between individuals with cLBP and asymptomatic controls. (**B**) Leave-one-out sensitivity analysis assessing the influence of individual studies on the pooled estimates of psoas major FI across lumbar levels

Three studies contributed data across four lumbar levels [26, 52, 54], with no statistically significant differences observed. Pooled effect estimates favoured higher psoas major fat infiltration (FI) in cLBP patients, except at L5/S1, where the effect was reversed (Fig. 10A). At upper-to-mid lumbar levels, exclusion of the Newell study [54] reversed the direction of effect in favour of greater FI in controls, though results remained non-significant. At L5/S1, excluding Mahmoudi’s instability subgroup [52] yielded a significant negative effect (SMD = − 1.23; 95% CI: − 2.43 to − 0.04), indicating higher FI in controls (Fig. 10B).

## Discussion

### Overview and study aims

cLBP remains the leading global cause of disability, yet its underlying musculoskeletal mechanisms are not fully understood. Increasing attention has been directed toward the paraspinal muscles—particularly the multifidus, erector spinae, and psoas major—due to their essential role in spinal stability and mounting evidence from animal and human studies linking cLBP with altered myoelectric activity and structural muscle changes, including atrophy, fat infiltration, and fibre type alterations [30, 57, 58]. This systematic review and meta-analysis aimed to determine whether individuals with cLBP exhibit consistent differences in paraspinal muscle morphology and composition compared to asymptomatic controls. By stratifying analyses by vertebral level and outcome—tCSA, fCSA, and FI—we sought to identify segment-specific differences that may hold clinical relevance.

### Principal findings

This meta-analysis shows that cLBP is associated with localised degeneration in the multifidus muscle, particularly at the mid-to-lower lumbar levels (L3/L4 to L5). Specifically, individuals with cLBP showed lower tCSA and fCSA, alongside significantly higher fat infiltration (FI)—a pattern consistent with prior systematic reviews [27, 30–32]. The effect sizes for these differences were generally in the small-to-moderate range, though some multifidus outcomes, such as tCSA at L4 and FI at L3/L4, reached large effects (< 0.8).

The multifidus FI may be linked to inflammatory processes characteristic of degenerative spine conditions. Supporting this, experimental models in both humans and animals, have reported increased macrophage infiltration and elevated tumour necrosis factor levels in the multifidus, suggesting a potential inflammatory mechanism underlying changes in muscle composition [59–61]. Moreover, the segment-specific nature of these alterations may reflect the unique biomechanical demands placed on the lower lumbar spine, which endures greater mechanical loading due to its proximity to the fixed pelvis and central role in load transmission [62]. Conversely, changes in the morphology and composition of the erector spinae and psoas major were less consistent, with statistically significant differences observed only at specific vertebral levels or identified through sensitivity analyses, indicating more variability in their involvement in cLBP.

The predominant involvement of the multifidus in cLBP is well-supported by its anatomical configuration and functional role. As a key deep segmental stabiliser, the multifidus is essential for maintaining spinal stiffness within the neutral zone, the range of intervertebral motion around the neutral posture where passive structures provide little resistance and active muscular control predominates. Within this range, the multifidus contributes up to two-thirds of active stabilisation [63]. Its short fibre length, high cross-sectional area, and tightly packed fascicles permit the generation of large stabilising forces over a short operating range, an ideal design for segmental control rather than gross spinal motion [64]. Functionally, the multifidus regulates vertebral rotation and axial load transmission, thereby facilitating coordinated spinal mechanics and protecting adjacent structures [65]. Progressive loss of multifidus muscle mass disrupts these functions, predisposing individuals to spinal instability, chronic pain, and functional impairment [66].

Mechanistically, these findings align with Panjabi’s stabilisation model, which frames spinal stability as the interaction of passive, active, and neural subsystems [67]. The multifidus, as the principal segmental stabiliser, is especially vulnerable to dysfunction in the active and neural domains. Arthrogenic muscle inhibition, characterised by a reflexive reduction in neural drive in response to pain, has been documented in the multifidus through electromyography and ultrasound, leading to impaired activation [41, 68]. Combined with cortical reorganisation in chronic pain states, this impaired control helps explain why multifidus degeneration often persists beyond the resolution of acute symptoms and contributes to recurrent pain [69].

### Sensitivity analyses and mechanistic interpretations

These structural and compositional differences in the multifidus were further elucidated through sensitivity analyses, which revealed the extent to which pooled effects were influenced by study-level demographic and methodological factors. For instance, although Liu’s [50] large, demographically balanced cohort helped reduce heterogeneity at several levels, its inclusion often attenuated effect size, potentially obscuring significant between-group differences. In contrast, the Newell study [54], characterised by control participants who were markedly older and leaner than those with cLBP, exerted a disproportionate influence on pooled estimates, frequently altering the direction of the effect and amplifying group differences. Exclusion of the Sions study [44] at L5 of the multifidus tCSA, which included participants with a mean age exceeding 70 years, unmasked significant multifidus atrophy in cLBP patients, underscoring the confounding role of age.

Similar patterns emerged in the analysis of multifidus FI, with significant increases in FI at L3/L4 and L4/L5 among individuals with cLBP being highly sensitive to study inclusion. Notably, at L4/L5, exclusion of Mahmoodi’s subgroup without lumbar segmental instability [52] rendered the effect non-significant, suggesting that spinal instability may facilitate localised fat infiltration through disrupted neuromuscular control. At L5/S1, removal of the Newell dataset [54] produced a significant effect and markedly reduced heterogeneity, reinforcing the importance of demographic matching.

In contrast, no statistically significant differences were observed in the erector spinae for either tCSA or fCSA, although pooled point estimates tended to favour slightly larger muscle size in cLBP patients, particularly at the lower lumbar levels. These trends may reflect compensatory hypertrophy in response to segmental instability or altered load-sharing dynamics, but were not robust across analyses, and between-study heterogeneity remained substantial. For erector spinae FI, no significant differences emerged, neither in primary analyses nor in sensitivity analysis. This pattern aligns with the erector spinae’s primary function as global stabilisers responsible for resisting anterior flexion and maintaining postural alignment [70]. As global muscles, they may be less susceptible to the focal arthrogenic inhibition affecting deep segmental stabilisers.

The psoas major which plays a dynamic role in trunk stabilisation and operates synergistically with posterior muscles to maintain sagittal alignment and lumbar balance [71], displayed a more variable profile. A significant reduction in tCSA was found only at L2/L3, though this was contingent upon the inclusion of Mahmoodi’s subgroup with lumbar instability [52], highlighting the potential influence of mechanical dysfunction on muscle morphology. At L3/L4, exclusion of Arbanas’ study [42], which featured an older participant cohort, revealed a significant reduction in muscle size among cLBP patients and eliminated heterogeneity, suggesting age-related bias may have previously masked true effects. Functional CSA differences in the psoas major were limited to L3/L4, yet again were sensitive to the inclusion of the Newell dataset [54]. Although no significant differences in FI were observed overall, sensitivity analysis at L5/S1 revealed a significant effect favouring greater FI in controls upon removal of Mahmoodi’s non-instability subgroup [52], raising the possibility of divergent muscle adaptation patterns under distinct clinical subtypes of cLBP.

### Methodological considerations

Several methodological factors likely contributed to the observed heterogeneity and sensitivity in pooled effect estimates. Variability in imaging modality, segmentation technique, and measurement protocol may have introduced inconsistencies in muscle quantification across studies. Moreover, participant-level characteristics—including sex distribution, age, BMI, and sample size—varied across studies and may have influenced outcomes. For example, the relatively lean cLBP cohort in Liu’s study [50] contrasted with the much higher BMI observed in Mahmoodi’s [52] and Newell’s cohorts [54], potentially affected estimates of muscle mass and fat infiltration. This is supported by findings from a large Chinese study (*n* = 516 females), which reported significantly greater FI in obese individuals compared to those with normal BMI across all adult age groups, while also identifying positive correlations between BMI and both muscle and intermuscular adipose tissue CSA [72]. Similarly, the inclusion of older populations, such as in the studies by Sions [44] and Arbanas [42], may have introduced confounding age-related degenerative changes due to sarcopenia (characterised by progressive loss of muscle fibres), thereby obscuring the specific effects of cLBP [73]. Importantly, none of the studies reported participant ethnicity or physical activity levels, both of which are known to influence muscle morphology and may play a role in the onset and persistence of cLBP [74, 75].

### Strengths and limitations

This is the first systematic review and meta-analysis to assess lumbar-level-specific morphology and composition of the multifidus, erector spinae, and psoas major muscles in cLBP. This work provides clinically relevant insights into the segmental nature of muscle degeneration in cLBP. By excluding studies focused on acute or subacute LBP, specific spinal pathologies, athletes, and occupational cohorts, we aimed to minimise sample heterogeneity and ensure greater generalisability to the broader cLBP population. Quality assessment, performed using NOS in accordance with Cochrane guidelines, identified 20 of the 21 included studies as of good quality, supporting the internal validity of the findings.

Nonetheless, several limitations warrant consideration. High heterogeneity was observed in several analyses, partly attributable to variability in sample size, imaging modality, segmentation techniques, and study design. Despite increasing research interest in the role of paraspinal muscle morphology in cLBP, relatively few studies met the stringent inclusion criteria, limiting the overall statistical power and underscoring the need for additional high-quality investigations. This limited number of studies for each outcome variable in the subgroup meta-analyses (< 10) also precluded assessment of publication bias through funnel plots, as recommended by current methodological guidelines. Moreover, many studies evaluated muscle characteristics at only a single vertebral level, limiting the ability to detect consistent segmental patterns. Given that paraspinal muscles exhibit region-specific adaptations, future studies should avoid averaging values across levels and instead examine each vertebral level systematically.

A further limitation arises from the lack of level-specific pathology data. Most studies did not distinguish pathology types or consistently report lumbar abnormalities (e.g. disc degeneration, herniation, height loss, bulging). Consequently, observed muscle differences may reflect both cLBP and the spatial relationship between specific spinal pathologies and muscle adaptation. Future work should provide standardised reporting of co-existing pathologies to enable integrated analyses of structural, compositional and pathological factors.

The quality assessment process also warrants caution in interpretation. Although most studies were rated as high quality, this finding was somewhat unexpected given the known methodological variability in study design. The use of a modified NOS may have contributed to this outcome, although any inflation of scores would likely have been consistent across studies. Nonetheless, this underscores that risk of bias may not have been fully captured and that high-quality ratings should be interpreted conservatively.

The exclusive reliance on static imaging assessments in the included studies represents another significant limitation, given the functional role of the paraspinal muscles in both static and dynamic conditions. This methodological constraint limits our ability to capture neuromuscular control deficits that may be central to cLBP pathophysiology, and future research should integrate dynamic functional assessments alongside morphological measures. Nevertheless, biomarkers derived from static imaging remain clinically valuable for characterising structural muscle changes and informing diagnosis and management.

Demographic mismatch, particularly in age, sex, and BMI between cLBP and control groups, emerged as significant sources of bias in sensitivity analyses. Future studies should ensure better group matching and, where possible, stratify results by key demographic variables. Alternative study designs could also help mitigate these limitations, such as prospective longitudinal cohorts tracking muscle changes before and after cLBP onset, twin or family-based studies controlling for genetic factors, or within-subject designs comparing affected and unaffected sides in unilateral cLBP. Additionally, no included studies reported participants’ ethnicity or physical activity levels—factors that are likely to influence muscle morphology and may contribute to cLBP risk. Other potentially important confounders were also inconsistently addressed, including occupational physical demands, symptom duration and severity, and psychological factors such as depression. Even if not statistically adjusted for, we suggest that such information should be routinely reported to enhance the interpretability of findings and the utility of future evidence syntheses.

## Conclusion

This systematic review and meta-analysis highlight that individuals with cLBP exhibit segment-specific degeneration in the paraspinal musculature, with the multifidus muscle, particularly at mid-to-lower lumbar levels, showing the most consistent reductions in cross-sectional area and increases in fat infiltration. In contrast, changes in the erector spinae and psoas major were less consistent. The erector spinae showed no significant differences across outcomes, though non-significant trends suggest possible compensatory hypertrophy at lower lumbar levels. The psoas major exhibited level-dependent reductions in size that were highly sensitive to study inclusion and participant characteristics, indicating a more variable role in cLBP.

Taken together, these findings support the potential utility of multifidus morphology, especially fat infiltration and atrophy at key lumbar segments, as structural correlates associated with cLBP that may inform clinical assessment and therapeutic targeting. Emerging evidence also supports the role of targeted resistance-based interventions in reversing these degenerative changes, particularly at the most affected segments [75]. Further longitudinal studies are warranted to determine whether these structural alterations precede, follow, or develop concurrently with cLBP onset, and to optimise intervention strategies aimed at preserving or restoring spinal stability in cLBP populations.

## Supplementary Information

Below is the link to the electronic supplementary material.

Supplementary Material 1

Supplementary Material 2

## Data Availability

The authors confirm that the data supporting the findings of this study are available within the article and its supplementary materials.
