# Supplementary material for: Paraspinal muscle quality in chronic low back pain: a systematic review and meta-analysis of muscle atrophy and fat infiltration
Source: Eur Spine J. 2025 Oct 28;35(5):2693–711. doi: 10.1007/s00586-025-09454-z (PMC13241407; doi:10.1007/s00586-025-09454-z)
Supplement: Supplementary file 1 — Supplementary Material 1 [file 586_2025_9454_MOESM1_ESM.docx]

**Ovid MEDLINE(R) ALL 12/02/2025 - Total Papers identified = 535 (1979 – 2025)**

1. Low back pain.mp.

2. Low backache.mp.

3. low back pains.mp.

4. back pain, lower.mp.

5. sciatica.mp.

6. radiculopathy.mp.

7. lumbago.mp.

8. disc herniation.mp.

9. disk herniation.mp.

10. LBP.mp.

11. lower back pain.mp.

12. low back ache.mp.

13. back ache.mp.

14. Low Backache.mp.

15. Backache.af.

16. disc degeneration.mp.

17. Intervertebral Disc Degeneration.mp.

18. 1 or 2 or 3 or 4 or 5 or 6 or 7 or 8 or 9 or 10 or 11 or 12 or 13 or 14 or 15 or 16 or 17

19. paraspinal muscles.mp.

20. vertebral muscle.mp.

21. paravertebral muscles.mp.

22. erector spinae.mp.

23. multifidus.mp.

24. psoas.mp.

25. 19 or 20 or 21 or 22 or 23 or 24

26. 18 and 25

27. muscular atrophy.mp.

28. muscle atrophy.mp.

29. muscle size.mp.

30. muscle morphology.mp.

31. paraspinal muscle atrophy.mp.

32. cross-sectional area.mp.

33. neurogenic muscular atrophy.mp.

34. muscular size.mp.

35. csa.mp.

36. muscle thickness.mp.

37. muscular thickness.mp.

38. fat infiltration.mp.

39. fatty infiltration.mp.

40. fat deposition.mp.

41. intramuscular fat.mp.

42. fat tissue.mp.

43. fatty tissue.mp.

44. adipose tissue.mp.

45. fat.mp.

46. muscle structure.mp.

47. muscular structure.mp.

48. muscular morphology.mp.

49. muscle composition.mp.

50. muscular composition.mp.

51. 27 or 28 or 29 or 30 or 31 or 32 or 33 or 34 or 35 or 36 or 37 or 38 or 39 or 40 or 41 or 42 or 43 or 44 or 45 or 46 or 47 or 48 or 49 or 50

52. 26 and 51

53. magnetic resonance imaging.mp.

54. MRI.mp.

55. Tomography.mp.

56. X-Ray Computed.mp.

57. CT scan.mp.

58. Ultrasound.mp.

59. dixon scan.mp.

60. dixon method.mp.

61. dixon technique.mp.

62. 53 or 54 or 55 or 56 or 57 or 58 or 59 or 60 or 61

63. 52 and 62

**Pubmed 12/02/2025 - (147 studies)**

((((("LBP"[Title/Abstract] OR "Low back pain"[Title/Abstract] OR ("back pain"[All Fields] AND "Low"[All Fields]) OR ("Pain"[Title/Abstract] AND "Low Back"[Title/Abstract]) OR ("Low back pain"[MeSH Terms] OR ("Low"[All Fields] AND "back"[All Fields] AND "Pain"[All Fields]) OR "Low back pain"[All Fields] OR "lumbago"[All Fields]) OR "Lower Back Pain"[All Fields] OR ("back pain"[Title/Abstract] AND "Lower"[Title/Abstract]) OR (("Pain"[MeSH Terms] OR "Pain"[All Fields]) AND "Lower Back"[All Fields]) OR "Low Back Ache"[All Fields] OR (("Pain"[MeSH Terms] OR "Pain"[All Fields] OR "ache"[All Fields]) AND "Low Back"[All Fields]) OR ("Back Ache"[All Fields] AND "Low"[All Fields]) OR "Low Backache"[All Fields] OR (("back pain"[MeSH Terms] OR ("back"[All Fields] AND "Pain"[All Fields]) OR "back pain"[All Fields] OR "backache"[All Fields] OR "backaches"[All Fields]) AND "Low"[All Fields]) OR "back pain"[All Fields] OR (("Pain"[MeSH Terms] OR "Pain"[All Fields]) AND ("back"[MeSH Terms] OR "back"[All Fields])) OR ("back pain"[MeSH Terms] OR ("back"[All Fields] AND "Pain"[All Fields]) OR "back pain"[All Fields] OR "backache"[All Fields] OR "backaches"[All Fields]) OR "Back Ache"[All Fields] OR (("Pain"[MeSH Terms] OR "Pain"[All Fields] OR "ache"[All Fields]) AND ("back"[MeSH Terms] OR "back"[All Fields])) OR "lumbago"[All Fields] OR "disc herniation"[All Fields] OR "Intervertebral Disc Degeneration"[All Fields]) AND "muscular atrophy"[All Fields]) OR (("atrophie"[All Fields] OR "atrophy"[MeSH Terms] OR "atrophy"[All Fields] OR "atrophied"[All Fields] OR "atrophies"[All Fields] OR "atrophying"[All Fields]) AND "muscular"[All Fields]) OR ("atrophy"[Title/Abstract] AND "muscle"[Title/Abstract]) OR "muscle atrophy"[All Fields] OR "neurogenic muscular atrophy"[All Fields] OR (("atrophie"[All Fields] OR "atrophy"[MeSH Terms] OR "atrophy"[All Fields] OR "atrophied"[All Fields] OR "atrophies"[All Fields] OR "atrophying"[All Fields]) AND "neurogenic muscular"[All Fields]) OR ("muscular atrophy"[All Fields] AND ("neurogenic"[All Fields] OR "neurogenically"[All Fields] OR "neurogenics"[All Fields])) OR "neurogenic muscular atrophy"[All Fields] OR (("atrophie"[All Fields] OR "atrophy"[MeSH Terms] OR "atrophy"[All Fields] OR "atrophied"[All Fields] OR "atrophies"[All Fields] OR "atrophying"[All Fields]) AND (("neurotrophic"[All Fields] OR "neurotrophics"[All Fields] OR "neurotrophism"[All Fields]) AND "muscular"[All Fields])) OR ("muscular atrophy"[All Fields] AND ("neurotrophic"[All Fields] OR "neurotrophics"[All Fields] OR "neurotrophism"[All Fields])) OR "muscle size"[All Fields] OR "muscular size"[All Fields] OR "cross sectional area"[All Fields] OR "csa"[Title/Abstract] OR "muscular size"[All Fields] OR "muscle thickness"[All Fields] OR "muscular thickness"[All Fields] OR "fat infiltration"[All Fields] OR "fatty infiltration"[All Fields] OR "fat deposition"[All Fields] OR "intramuscular fat"[All Fields] OR "fat tissue"[All Fields] OR "fatty tissue"[All Fields] OR "adipose tissue"[All Fields] OR "fat"[All Fields] OR "muscle structure"[Title/Abstract] OR "muscular structure"[Title/Abstract] OR "muscle morphology"[All Fields] OR (("anatomy and histology"[MeSH Subheading] OR ("anatomy"[All Fields] AND "histology"[All Fields]) OR "anatomy and histology"[All Fields] OR "morphology"[All Fields] OR "morphologies"[All Fields]) AND ("muscle s"[All Fields] OR "muscles"[MeSH Terms] OR "muscles"[All Fields] OR "muscle"[All Fields])) OR "muscular morphology"[All Fields] OR "muscle composition"[All Fields] OR "muscular composition"[All Fields]) AND "paraspinal muscles"[Title/Abstract]) OR "paraspinal muscles"[All Fields] OR "erector spinae"[All Fields] OR "multifidus"[All Fields] OR "psoas"[All Fields]) AND ((observationalstudy[Filter]) AND (humans[Filter]) AND (1900:2025[pdat]) AND (english[Filter]) AND (alladult[Filter]))

**Scopus – 12/02/2025 – 1443 publications**

( TITLE-ABS ( lbp ) OR TITLE-ABS ( "Low back pain" ) OR ( TITLE-ABS ( "Back Pain" ) AND TITLE-ABS ( low ) ) OR ( TITLE-ABS ( pain ) AND TITLE-ABS ( "Low Back" ) ) OR TITLE-ABS ( lumbago ) OR TITLE-ABS ( "Lower Back Pain" ) OR ( TITLE-ABS ( "Back Pain" ) AND TITLE-ABS ( lower ) ) OR ( TITLE-ABS ( pain ) AND TITLE-ABS ( "Lower Back" ) ) OR TITLE-ABS ( "Low Back Ache" ) OR ( TITLE-ABS ( ache ) AND TITLE-ABS ( "Low Back" ) ) OR ( TITLE-ABS ( "Back Ache" ) AND TITLE-ABS ( low ) ) OR ALL ( "Low Backache" ) OR ( ALL ( backache ) AND ALL ( low ) ) OR ALL ( "Back pain" ) OR ( ALL ( pain ) AND ALL ( back ) ) OR TITLE-ABS ( backache ) OR TITLE-ABS ( "Back Ache" ) OR ( TITLE-ABS ( ache ) AND TITLE-ABS ( back ) ) OR TITLE-ABS ( disc AND herniation ) OR ALL ( "Intervertebral Disc Degeneration" ) ) AND ( TITLE ( "muscular atrophy" ) OR ( TITLE ( atrophy ) AND TITLE ( muscular ) ) OR ( TITLE-ABS ( atrophy ) AND TITLE-ABS ( muscle ) ) OR TITLE-ABS ( "muscle atrophy" ) OR TITLE-ABS ( "neurogenic muscular atrophy" ) OR ( ALL ( atrophy ) AND ALL ( "neurogenic muscular" ) ) OR ( TITLE-ABS ( "muscular atrophy" ) AND TITLE-ABS ( neurogenic ) ) OR ALL ( "neurogenic muscular atrophy" ) OR ( ALL ( atrophy ) AND ALL ( "neurotrophic muscular" ) ) OR ( ALL ( "muscular atrophy" ) AND ALL ( neurotrophic ) ) OR TITLE-ABS ( "muscle size" ) OR TITLE-ABS ( "muscular size" ) OR TITLE-ABS ( "cross sectional area" ) OR TITLE-ABS ( csa ) OR TITLE ( "muscular size" ) OR TITLE ( "muscle thickness" ) OR ALL ( "muscular thickness" ) OR ALL ( "fat infiltration" ) OR TITLE-ABS ( "fatty infiltration" ) OR ALL ( "fat deposition" ) OR TITLE ( "intramuscular fat" ) OR ALL ( "fat tissue" ) OR TITLE-ABS ( "fatty tissue" ) OR TITLE ( "adipose tissue" ) OR TITLE-ABS ( fat ) OR TITLE-ABS ( "muscle structure" ) OR TITLE-ABS ( "muscular structure" ) OR TITLE ( "muscle morphology" ) OR ( TITLE ( morphology ) AND TITLE ( muscle ) ) OR ALL ( "muscular morphology" ) OR TITLE ( "muscle composition" ) OR TITLE ( "muscular composition" ) ) AND ( ALL ( "paraspinal muscles" ) OR ALL ( "erector spinae" ) OR ALL ( "multifidus" ) OR ALL ( "psoas" ) ) AND ( ALL ( "magnetic resonance imaging" ) OR ALL ( mri ) OR ALL ( tomography ) OR ALL ( "X-Ray Computed" ) OR ALL ( "CT scan" ) OR ALL ( ultrasound ) OR ALL ( "dixon scan" ) OR ALL ( "dixon method" ) OR ALL ( "dixon technique" ) ) AND ( EXCLUDE ( DOCTYPE , "re" ) OR EXCLUDE ( DOCTYPE , "cp" ) OR EXCLUDE ( DOCTYPE , "ch" ) OR EXCLUDE ( DOCTYPE , "bk" ) OR EXCLUDE ( DOCTYPE , "no" ) OR EXCLUDE ( DOCTYPE , "le" ) OR EXCLUDE ( DOCTYPE , "sh" ) OR EXCLUDE ( DOCTYPE , "tb" ) OR EXCLUDE ( DOCTYPE , "ed" ) ) AND ( LIMIT-TO ( LANGUAGE , "English" ) ) AND ( EXCLUDE ( EXACTKEYWORD , "Surgery" ) OR EXCLUDE ( EXACTKEYWORD , "Young Adult" ) OR EXCLUDE ( EXACTKEYWORD , "Adolescent" ) OR EXCLUDE ( EXACTKEYWORD , "Nonhuman" ) OR EXCLUDE ( EXACTKEYWORD , "Animals" ) )

**Embase Classic+Embase - 12/02/2025 – 1005 studies**

1. Low back pain.mp.

2. Low backache.mp.

3. low back pains.mp.

4. back pain, lower.mp.

5. sciatica.mp.

6. radiculopathy.mp.

7. lumbago.mp.

8. disc herniation.mp.

9. disk herniation.mp.

10. LBP.mp.

11. lower back pain.mp.

12. low back ache.mp.

13. back ache.mp.

14. Low Backache.mp.

15. Backache.af.

16. disc degeneration.mp.

17. Intervertebral Disc Degeneration.mp.

18. 1 or 2 or 3 or 4 or 5 or 6 or 7 or 8 or 9 or 10 or 11 or 12 or 13 or 14 or 15 or 16 or 17

19. paraspinal muscles.mp.

20. vertebral muscle.mp.

21. paravertebral muscles.mp.

22. erector spinae.mp.

23. multifidus.mp.

24. psoas.mp.

25. 19 or 20 or 21 or 22 or 23 or 24

26. 18 and 25

27. muscular atrophy.mp.

28. muscle atrophy.mp.

29. muscle size.mp.

30. muscle morphology.mp.

31. paraspinal muscle atrophy.mp.

32. cross-sectional area.mp.

33. neurogenic muscular atrophy.mp.

34. muscular size.mp.

35. csa.mp.

36. muscle thickness.mp.

37. muscular thickness.mp.

38. fat infiltration.mp.

39. fatty infiltration.mp.

40. fat deposition.mp.

41. intramuscular fat.mp.

42. fat tissue.mp.

43. fatty tissue.mp.

44. adipose tissue.mp.

45. fat.mp.

46. muscle structure.mp.

47. muscular structure.mp.

48. muscular morphology.mp.

49. muscle composition.mp.

50. muscular composition.mp.

51. 27 or 28 or 29 or 30 or 31 or 32 or 33 or 34 or 35 or 36 or 37 or 38 or 39 or 40 or 41 or 42 or 43 or 44 or 45 or 46 or 47 or 48 or 49 or 50 52. 26 and 51
